# Anticancer Activity of Chamaejasmine: Effect on Tubulin Protein

**DOI:** 10.3390/molecules16086243

**Published:** 2011-07-25

**Authors:** Wenlong Fang, Songtao Liu, Yingkun Nie

**Affiliations:** 1 Department of Rheumatology, The Second Hospital Affiliated Harbin Medical University, Harbin 150086, China; Email: Wenlong@yahoo.com.cn (W.F.); 2 Hei longjiang Disabled Federation for Human Care Clinic, Harbin 150020, China; Email: liusongtao@126.com (S.L.)

**Keywords:** chamaejasmine, anti-cancer activity, PC-3 cells, tubulin, docking

## Abstract

In this work, the anticancer activity of chamaejasmine was studied by evaluating its *in vitro* cytotoxicity against several human cancer cell lines (MCF-7, A549, SGC-7901, HCT-8, HO-4980, Hela, HepG2, PC-3, LNCap, Vero and MDCK) using the MTT assay. Results indicated chamaejasmine showed more notable anticancer activity than taxol against PC-3 cells, with IC_50_ values of 2.28 and 3.98 µM, respectively. Furthermore, Western blot analysis showed that chamaejasmine was able to increase the expression of β-tubulin, but not α-tubulin. *In silico* simulations indicated that chamaejasmine specifically interacts with the active site which is located at the top of β-tubulin, thanks to the presence of strong hydrophobic effects between the core templates and the hydrophobic surface of the TB active site. The binding energy (E*_inter_*) was calculated to be −164.77 kcal·mol^−1^. Results presented here suggest that chamaejasmine possesses anti-cancer properties relating to β-tubulin depolymerization inhibition, and therefore is a potential source of anticancer leads for the pharmaceutical industry.

## 1. Introduction

Cancer, which is a great threat to human life, is a serious disease with a complex pathogenesis. Many chemotherapeutic drugs have been developed to treat cancer, including DNA-alkylating agents and antimitotic agents [1,2]. A great number of natural products such as taxol and vinca alkaloids are well known for their effective antimitotic activity. However, their complex synthesis, difficult formulation, and lack of oral availability makes these drugs suboptimum for clinical treatment of cancer [3]. Hence, there is considerable interest in the development of novel molecules that inhibit tubulin polymerization.

In the past three decades, microtubules continue to be one of the most successful cancer chemotherapeutic targets [4,5,6,7,8]. The taxenes and vinca alkaloids are well-characterized anti-mitotic compounds which are widely used in clinical situations. However, the development of multidrug resistance has limited the potency of these antimitotic compounds [9]. Therefore, there has been a great interest in identifying novel microtubule inhibitors that can overcome various modes of resistance and exhibit improved pharmacology profiles [10,11,12].

**Figure 1:**
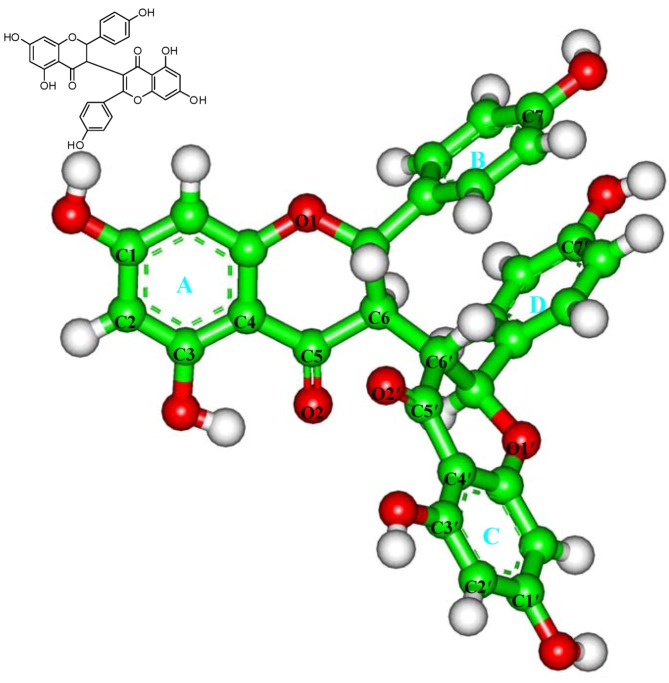
Structure of chamaejasmine.

Many medical plants have served as sources of anticancer pharmaceuticals, and over 60% of current anticancer drugs such as vinblastine, topotecan, etoposide, and paclitaxel were originally plant-derived compounds [13,14]. *Stellera chamaejasme* L., belongs to the Thymealaeaceae, widely distributed in northwest and southwest parts in China. The roots of *Stellera chamaejasme* L., can be used as pesticide on bugs, flies and maggots, and can also control pests on crops, and pastures [15,16]. It has also been found that the methanol extract of the root of *Stellera chamaejasme* L. showed significant antitumor activities [17]. Chamaejasmine (Figure 1) is a natural biflavanones with notable pesticidal activity [18]. However, the anticancer activity of chamaejasmine has not been elucidated yet.

In the present study, we first examined the growth inhibitory effect of chamaejasmine on MCF-7, A549, SGC-7901, HCT-8, HO-4980, Hela, HepG2, PC-3 and LNCap, Vero and MDCK cells by the [3-(4,5)-dimethylthiazoly1)-3,5-diphenytetrazolium bromide (MTT) assay. The expression of α-tubulin and β-tubulin proteins was further assayed by Western blotting. Furthermore, explicitly solvated flexible docking and molecular dynamic (MD) methods were applied to investigate the mechanisms of the inhibitory action of chamaejasmine on the tubulin protein. We anticipate that the insight gained into the binding mechanisms will be of value in the rational design of new anti-cancer agents.

## 2. Results and Discussion

### 2.1. Cytotoxicity Assays

The cytotoxicity of chamaejasmine was evaluated on nine human cancer cell lines (MCF-7, A549, SGC-7901, HCT-8, HO-4980, Hela, HepG2, PC-3 and LNCap) and two normal cell lines (Vero and MDCK) using MTT assays. Taxol was used as positive control. The results are listed in Table 1. As shown, chamaejasmine exhibited strong cytotoxicity against all nine cancer cell lines. Among all of them, chamaejasmine showed more notable cytotoxicity than taxol against MCF-7, HO-4980, A549 and PC-3, with IC_50_ values of 4.02, 5.31, 4.84 and 2.28 µM, respectively. Moreover, chamaejasmine showed less cytotoxicity than taxol against the Vero and MDCK lines. As shown in Table 2, chamaejasmine also reduced proliferation of PC-3 cells in a time-dependent manner. The inhibition action of 2 μM chamaejasmine appeared superior to taxol at 24 h, 48 h and 72 h.

**Table 1:** Effect of chamaejasmine towards MCF-7, A549, SGC-7901, HCT-8, HO-4980, Hela, Hep G2, PC-3, LNCap, Vero and MDCK cells as determined by the MTT assay.

**Cell Line**	**IC_50_ (µM)**	
	chamaejasmine	taxol
MCF-7	4.02	5.98
A549	4.84	6.18
SGC-7901	11.97	3.37
HCT-8	12.45	5.06
HO-4980	5.31	7.02
Hela	9.88	5.01
Hep G2	14.36	6.17
PC-3	2.28	3.98
LNCap	5.21	2.81
Vero	3.16	0.92
MDCK	4.57	0.83

**Table 2:** Effect of chamaejasmine towards PC-3 cells as determined by the MTT assay at different times.

**IC_50_ (µM)**	24 h	48 h	72 h
Chamaejasmine	10.52	5.05	2.28
Taxol	16.24	6.71	3.98

Many biflavones possess anti-tumor activity towards various human cancer cell lines, suggesting that they may be promising candidates for novel anticancer agents [19]. Amentoflavone, which was obtained from *Selaginella tamariscina*, showed certain anticancer activity against Hela and MCF-7, with IC_50_ values of 76.83 µM, 67.71 µM, respectively. It has a potential for cancer chemoprevention [19]. Chamaejasmine has a similar chemical structure, but lower IC_50_ values in the present investigation. Therefore, we have reason to believe that the potential of chamaejasmine in cancer therapy is more promising than that of amentoflavone.Because chamaejasmine showed its best antitumor activity towards PC-3, PC-3 was further used to study its effect on polymerized α-tubulin and β-tubulin.

### 2.2. Measurement of Tubulin Polymerization

**Figure 2:**
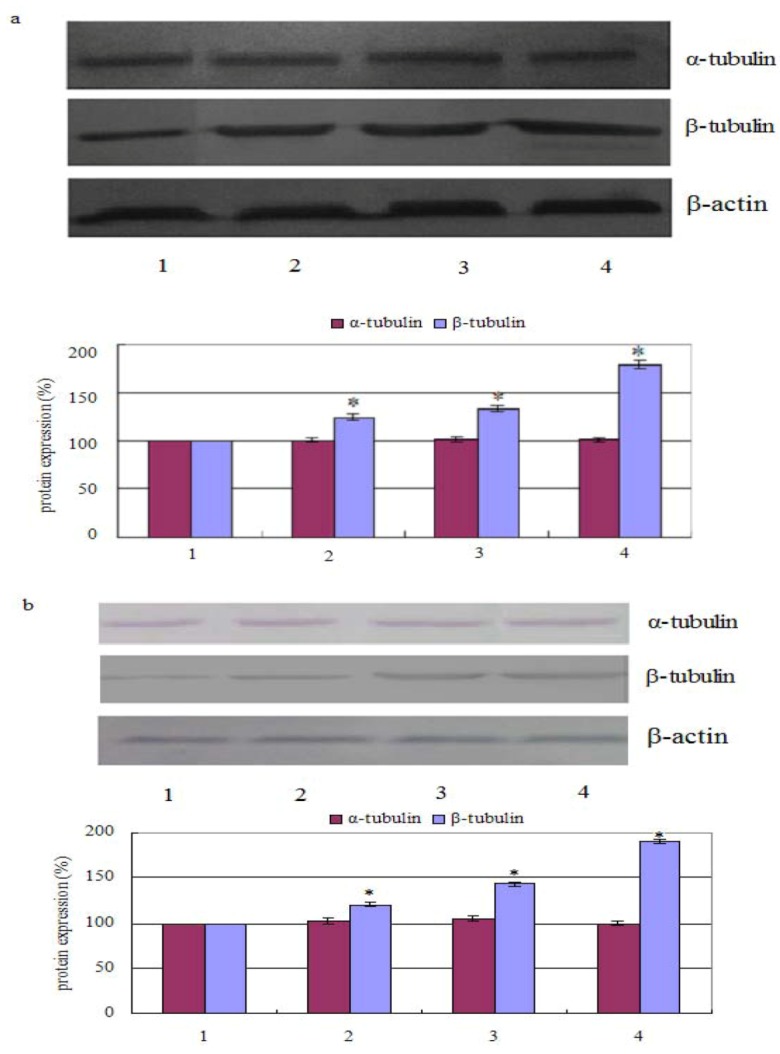
Effect of chamaejasmine on α-tubulin and β-tubulin activities by Western blotting assay, PC-3 cells were treated with chamaejasmine (0, 1, 2, 4 µM) for 72 h (**a**); PC-3 cells were treated with chamaejasmine for 0, 24, 48 and 72 h (**b**). * *P* < 0.05 compared with cell control.

The results showed that the percentage of polymerized β-tubulin before and after treatment with chamaejasmine changed from 124.21 ± 2.47%, 132.66 ± 2.98%, 178.57 ± 4.54% in PC-3 cells (in a concentration dependent manner). Whereas, after cells treated with 2 µM chamaejasmine, the percentage of polymerized β-tubulin increased to 120.64 ± 1.99%, 143.12 ± 2.26%, and 190.11 ± 3.02%, respectively. In contrast, the percentage of α-tubulin was largely unchanged with chamaejasmine treatment (Figure 2a,b).

In cells, microtubule filaments rapidly alternate between phases of growth and shrinkage (dynamic instability) during cell cycle. Since microtubules play crucial roles in the regulation of the mitotic apparatus, disruption of microtubules can induce cell cycle arrest in the M phase, leading to the formation of abnormal mitotic spindles and finally triggering of apoptosis signals [20].

### 2.3. *In Silico* Inhibition Mechanism of Chamaejasmine

**Figure 3:**
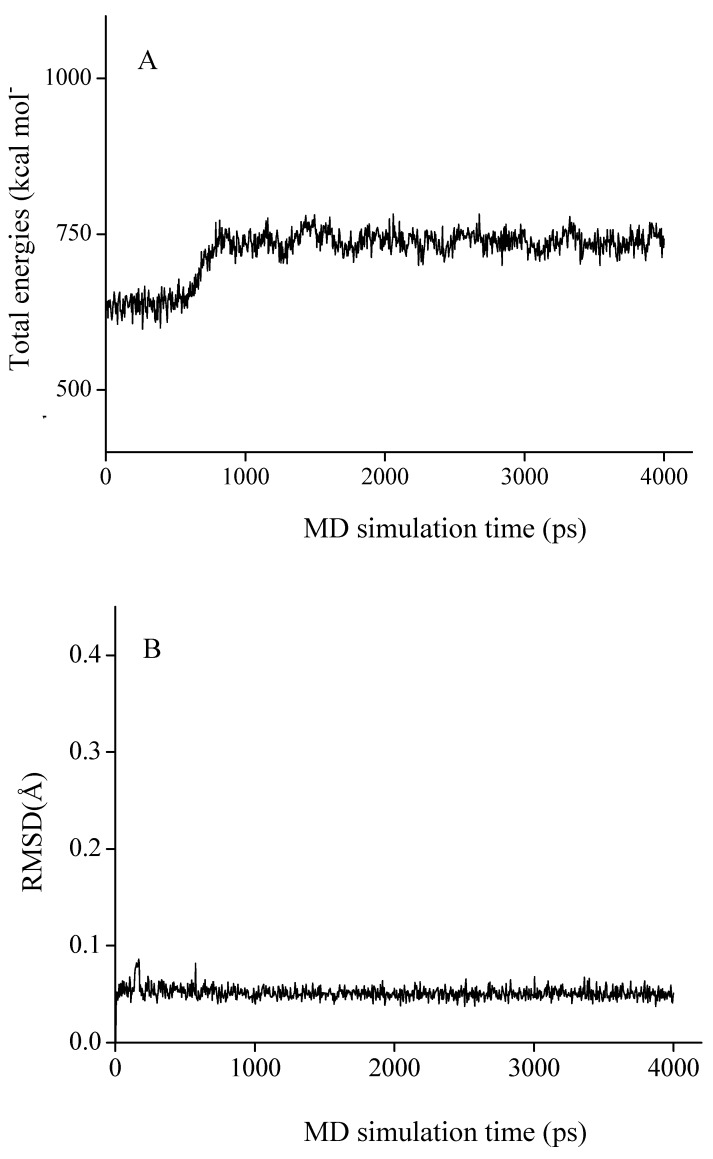
The time-evolution total energies (**A**) and backbone-atom root mean square deviations (RMSD, **B**) for the chamaejasmine-TB complex during the MD simulations.

Based on the results above, molecular docking and molecular dynamics (MD) studies were conducted to explore the inhibition mechanism of chamaejasmine. As the total energies and backbone root-mean-square-deviations (RMSD) in Figure 3 show, the docked complex quickly reaches equilibrium and remains rather stable after about 2.0 ns. Therefore, the average structure over the 2.0~4.0 ns MD trajectories was used for the further analysis.

**Figure 4:**
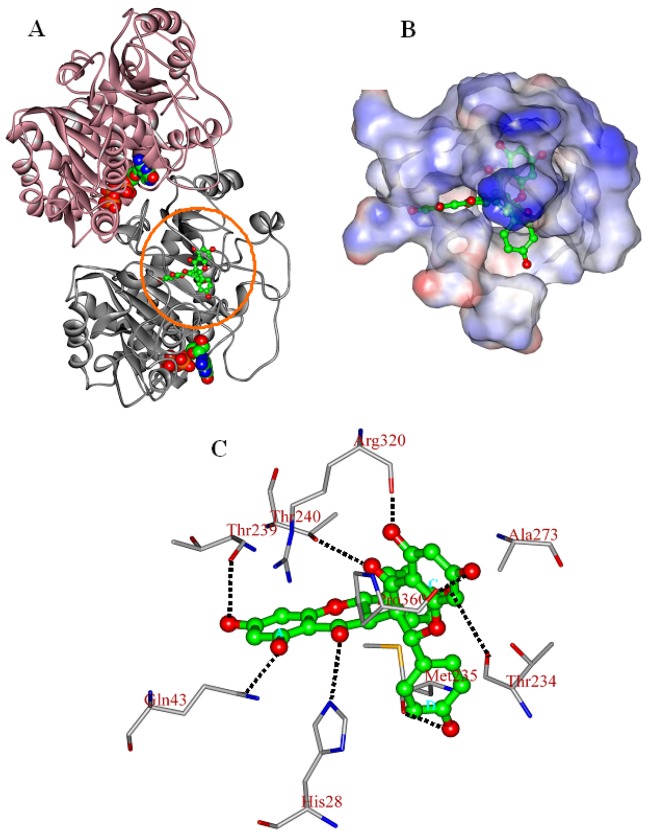
The propeller structure (**A**), the active site (**B**), and key residues in the active site of the chamaejasmine-TB complex. The tubulin (TB) protein is in ribbon. The gold ring shows the active site. The Connolly surfaces of the TB active site (in property) are created using the InsightII 2005scripts. Chamaejasmine is represented by the ball and stick model.

As shown in Figure 4A, chamaejasmine occupies the active site of tubulin (TB), which is mapped to the top of β-subunit of TB protein (β-TB). Its interaction energy (E*_inter_*) with TB protein is calculated to be −164.77 kcal·mol^−1^. The van der Walls interactions (E*_vdW_*) play a large role in the binding process, accounting for more than 67.5% of the total interaction energy (E*_inter_*). The binding pose of chamaejasmine within TB protein is characterized by the presence of strong hydrophobic effects between the core templates (phenyl rings A–D) and the hydrophobic surface of TB active site (Figure 4B). As shown in Figure 4C, the C1, C3, C7, C1′, C3′ and C7′ hydroxyl groups (–OH) of chamaejasmine are separately oriented towards residues ThrB239, GlnB43, ThrB234, ProB360, ArgB320 and MetB235, with one H-bond formed. Besides, the C5 and C5′carbonyl groups (–C=O) of chamaejasmine are bound towards residues HisB28 and ThrB240, with the formation of one H-bond, respectively. The eight strong H-bonds greatly introduce the inhibiting activity of chamaejasmine against the function of TB protein. Hereby, there are strong interactions (E*_sum_*) between chamaejasmine and the active-site residues HisB28, GlnB43, ThrB234, MetB235, ThrB239, AlaB273, ArgB320 and ProB360, especially residues ArgB320 and ProB360, where the values are calculated to be −24.20 and −13.71 kcal·mol^−1^, respectively (Table 3). In contrast to the situation of paclitaxel [21], chamaejasmine is more hydrophobically collapsed with the hydrophobic residues of β-TB and level off the docked complex, as a center of organization for TB active site through converting the hydrophobic cleft into a hydrophilic surface. As a result of this situation, the interaction energy (E*_inter_*) of chamaejasmine is higher than the value of paclitaxel (−127.96 kcal mol^−1^) [22], but the proportion of van der Walls effects is sharply reduced from 70.7% (paclitaxel) [22] to 67.5% (chamaejasmine). This indicated that the disassembly of αβ-TB protein may interfere with chamaejasmine and this effect is better than that of paclitaxel, consistent with the above experimental data [21,23].

Taken together, it is likely that chamaejasmine exerts its anti-tumor activities via inhibition of the β-TB protein disassembly. Therefore, it should be a potential lead compound in the developing the novel anti-tumor drugs. Further studies are urgently needed to support this point of view.

**Table 3:** The vdW, electrostatic and sum interaction energies (E*_vdW_*, E*_ele_* and E*_sum_*) involving chamaejasmine with the key residues of tubulin protein *^a^*.

Residue	E*_vdW_*	E*_ele_*	E*_sum_*
IleB24	−2.40	−0.60	−3.00
HisB28	−5.91	−3.13	−9.04
GlnB43	−1.51	−5.60	−7.11
ThrB234	−4.98	−3.95	−8.93
MetB235	−5.72	−4.35	−10.07
SerB236	−7.84	−1.14	−8.98
GlyB237	−5.51	−0.65	−6.16
ThrB239	−3.23	−1.99	−5.22
ThrB240	−6.64	−6.11	−12.75
PheB244	−3.79	0.53	−3.26
AlaB273	−2.52	−1.27	−3.79
ArgB320	−4.84	−19.36	−24.20
GlyB321	−1.63	−0.65	−2.28
ProB360	−5.54	−8.17	−13.71
ArgB369	−3.00	−0.23	−3.23
GlyB370	−3.77	0.62	−3.15
LeuB371	−3.11	−0.33	−3.44
SerB374	−8.22	−1.07	−9.29
AlaB375	−3.35	−0.04	−3.39

*^a^* Energy units in kcal mol^−1^.

## 3. Experimental

### 3.1. Materials

Chamaejasmine and taxol were obtained from Sigma Chemical Co. (St. Louis, MO, USA) and were stored in glass vials with Teflon sealed caps at −20 ± 0.5 °C in the absence of light. A 10 mM stock solution of chamaejasmine was prepared in dimethyl sulfoxide (DMSO) and stored at −80 °C. Deionized water was used in all experiments.

### 3.2. Growth of Cells

MCF-7, A549, SGC-7901, HCT-8, HO-4980, Hela, HepG2, PC-3, LNCap, Vero, MDCK cell lines were purchased from Harbin Medical University, China. All the tumor cells were maintained in Roswell Park Memorial Institute 1640 (RPMI 1640) medium supplemented with 10% fetal bovine serum and 100 U/mL penicillin and 100 μg/mL streptomycin. Vero and MDCK were maintained in DMEM medium supplemented with 10% fetal bovine serum and 100 U/mL penicillin and 100 μg/mL streptomycin. The cells were kept at 37 °C in a humidified atmosphere containing 5% CO_2_.

### 3.3. Cytotoxicity Assays

Inhibition of cell proliferation by chamaejasmine was measured by the MTT assay [19]. Briefly, cells were plated in 96-well culture plates (1 × 10^5^ cells/well) separately. After 24 h incubation, cells were treated with chamaejasmine (0, 3.13, 6.25, 12.5, 25, 50 and 100 μM, eight wells per concentration) for 72 h, MTT solution (5 mg/mL) was then added to each well. After 4 h incubation, the formazan precipitate was dissolved in 100 μL dimethyl sulfoxide, and then the absorbance was measured in an ELISA reader (Thermo Molecular Devices Co., Union City, CA, USA) at 570 nm. The cell viability ratio was calculated by the following formula: Inhibitory ratio (%) = (ODcontrol − ODtreated)/ODcontrol × 100%. Cytotoxicity was expressed as the concentration of chamaejasmine inhibiting cell growth by 50% (IC_50_ value).

### 3.4. Western Blot Assay

PC-3 cells were treated with chamaejasmine (0, 0.5, 2 and 4 µM) for 72 h or with 2 µM chamaejasmine for 0, 24, 48 and 72 h. For isolation of total protein fractions, cells were collected, washed twice with ice-cold PBS, and lysed using cell lysis buffer [20] mM Tris pH 7.5, 150 mM NaCl, 1% Triton X-100, 2.5 mM sodium pyrophosphate, 1 mM EDTA, 1% Na_3_CO_4_, 0.5 μg/mL leupeptin, 1 mM phenylmethanesulfonyl fluoride (PMSF)]. The lysates were collected by scraping from the plates and then centrifuged at 10,000 rpm at 4 °C for 5 min.

Total protein samples (20 μg) were loaded on a 12% of SDS-polyacrylamide gel for electrophoresis, and transferred onto PVDF transfer membranes (Millipore, Billerica, MA, USA) at 0.8 mA/cm^2^ for 2 h. Membranes were blocked at room temperature for 2 h with blocking solution (1% Bovine Serum Albumin (BSA) in Phosphate Buffered Saline (PBS) plus 0.05% Tween-20). Membranes were incubated overnight at 4 °C with primary antibodies (anti-β-actin was mouse polyclonal antibodies; anti-α-tubulin and anti-β-tubulin, were rabbit polyclonal antibodies) at a 1:1000 dilution (Biosynthesis Biotechnology Company, Beijing, China) in blocking solution. After thrice washings in Tris Buffer Solution Tween (TBST) for each 5 min, membranes were incubated for 1 h at room temperature with an alkaline phosphatase peroxidase-conjugated anti-mouse secondary antibody at a dilution of 1:500 in blocking solution. Detection was performed by the BCIP/NBT Alkaline Phosphatase Color Development Kit (Beyotime Institute of Biotechnology) according to the manufacturer’s instructions. Bands were recorded by a digital camera (Canon EOS 350D, Tokyo, Japan).

### 3.5. *In Silico* Simulation

The docking and molecular dynamics (MD) simulations were performed with the different modules of InsightII 2005 software package on Linux workstations, using the consistent-valence force-field (CVFF) [24]. The CVFF force-field was widely used in the ligand-protein interacting systems [25,26,27,28,29,30,31], including the tubulin-related ones [25,26].

#### 3.5.1. System preparations

The atomic coordinates of the αβ-tubulin dimer were obtained from the PDB database (PDB Accession number: 1JFF) [23]. For convenience, the structure is named as TB subsequently. The substrate (paclitaxel) and heteroatoms were removed from the crystal structure, following by the incorrect bonds manually modified (Biopolymer module) [25,32,33]. The hydrogen atoms were added to the TB protein on basis of the expected charge distribution of amino acids at physiological pH [32,33]. Then, the TB structure was solvated in a sphere of the TIP3P water molecules [34] with the radius of 35.0 Å, which is large enough to contain the assembly. The Na^+^ ions were added to neutralize the system [35]. The resulting system was first minimized for 500 steps with steep decent (SD) method and then optimized using the conjugate gradient (CG) method with the convergence criterion of 0.01 kcal·mol^−1^·Å^−1^. The atomic charge and structural parameters of chamaejasmine (Figure 1) were derived using the BFGS algorithm (Discover 3.0 module) [24].

#### 3.5.2. Docking

The active site of TB protein was identified with the aid of Binding-site module [21,23]. With the aid of Affinity module, the chamaejasmine was docked into the active site of TB protein. Its optimal orientation at the TB active site was determined with the combining of Monte Carlo (MC) type and simulation annealing (SA) methods [36]. The solvent effects were considered by solvating the complexes in a large sphere of TIP3P water [34]. The non-bonded interactions were considered by the Cell-Multipole approach. The selected complex was further energy-minimized using the conjugated gradient method until converged to 0.01 kcal·mol^−1^·Å^−1^. The CVFF force-field was used for the optimizations. For more details about the calculations readers are referred elsewhere [30,31].

#### 3.5.3. Molecular dynamics (MD)

A 4.0-ns MD simulation was performed on the energy-minimized chamaejasmine -TB complex by Discover 3.0 module, using the CVFF force-field. The canonical ensemble (NVT) was employed and the non-bonded interactions were calculated by the Cell-Multipole approach. The velocity scaling thermostat was used for the temperature controls, which allows the MD simulation temperatures to change within a small range around the defined value (300K) [37]. Integrations of the classical equations of motion were achieved using the Verlet algorithm with an integration step of 2.0 fs. All atoms within 6.0 Å of the ligand were allowed to move freely during the MD simulations, whereas those atoms beyond 6.0 Å were held rigid [38]. The MD trajectories were saved every 4,000 steps. The geometric and energetic analyses were based on the average structure over the 2.0~4.0 ns MD trajectories.

### 3.6. Statistical Analysis

The Student’s t-test was used to compare the difference between two different groups. A value of *p* < 0.05 was considered to be statistically significant.

## 4. Conclusions

The current study demonstrated the anticancer activity of chamaejasmine as well as the inhibition of binding process between α-tubulin, β-tubulin and chamaejasmine. Further pharmacological investigations are necessary to provide firm evidence about the anticancer mechanism of chamaejasmine.

## References

[1] Coxon A., Bush T., Saffran D., Kaufman S., Belmontes B., Rex K., Hughes P., Caenepeel S., Rottman J.B., Tasker A. (2009). Broad antitumor activity in breast cancer xenografts by motesanib, a highly selective, oral inhibitor of vascular endothelial growth factor, platelet-derived growth factor, and Kit receptors. Clin. Cancer Res..

[2] Munroe M.E., Arbiser J.L., Bishop G.A. (2007). Honokiol, a natural plant product, inhibits inflammatory signals and alleviates inflammatory arthritis. J. Immunol..

[3] Kavallaris M., Verrills N.M., Hill B.T. (2001). Anticancer therapy with novel tubulin-interacting drugs. Drug Resist. Updat..

[4] Shi Q., Chen K., Morris-Natschke S.L., Lee K.H. (1998). Recent progress in the development of tubulin inhibitors as antimitotic antitumor agents. Curr. Pharm. Des..

[5] Jordan A., Hadfield J.A., Lawrence N.J., McGown A.T. (1998). Tubulin as a target for anticancer drugs: agents which interact with the mitotic spindle. Med. Res. Rev..

[6] Islam M.N., Iskander M.N. (2004). Microtubulin binding sites as target for developing anticancer agents. Mini Rev. Med. Chem..

[7] Jordan M.A., Wilson L. (2004). Microtubules as a target for anticancer drugs. Nat. Rev. Cancer.

[8] Teicher B.A. (2008). Newer cytotoxic agents: Attacking cancer broadly. Clin. Cancer Res..

[9] Dumontet C., Sikic B.I. (1999). Mechanisms of action of and resistance to antitubulin agents: microtubule dynamics, drug transport, and cell death. J. Clin. Oncol..

[10] Chang J.Y., Yang M.F., Chang C.Y., Chen C.M., Kuo C.C., Liou J.P. (2006). 2-amino and 2'-aminocombretastatin derivatives as potent antimitotic agents. J. Med. Chem..

[11] Chang J.Y., Hsieh H.P., Chang C.Y., Hsu K.S., Chiang Y.F., Chen C.M., Kuo C.C., Liou J.P. (2006). 7-Aroyl-aminoindoline-1-sulfonamides as a novel class of potent antitubulin agents. J. Med. Chem..

[12] Liou J.P., Wu C.Y., Hsieh H.P., Chang C.Y., Chen C.M., Kuo C.C., Chang J.Y. (2007). 4- and 5-aroylindoles as novel classes of potent antitubulin agents. J. Med. Chem..

[13] Newman D.J., Cragg G.M., Snader K.M. (2003). Natural products as sources of new drugs over the period 1981-2002. J. Nat. Prod..

[14] Cragg G.M., Newman D.J. (2005). Plants as a source of anti-cancer agents. J. Ethnopharmacol..

[15] Shi Z.C. (1997). The Poisonous Plants in Chinese Pasture.

[16] Zhao S.H., Wang S.Q. (1997). Progress of Investigations and Application of Insecticide Plants.

[17] Feng W., Tetsuro I., Mitsuzi Y. (1995). The antitumor activities of gnidimacrin isolated from Stellera chamaejasme L. Chin. J. Cancer Res..

[18] Tang X., Hou T. (2008). Development of chamaejasmin microemulsion and its biological activity against Aphis craccivora and Culex pipiens pallens. Flavour. Fragr. J..

[19] Jing Y., Zhang G.G., Ma E.L., Zhang H.M., Guan J., He J. (2010). Amentoflavone and the extracts from Selaginella tamariscina and their anticancer activity. Asian J. Traditional Med..

[20] Downing K.H. (2000). Structural basis for the interaction of tubulin with proteins and drugs that affect microtubule dynamics. Annu. Rev. Cell Dev. Biol..

[21] Snyder J.P., Nettles J.H., Cornett B., Downing K.H., Nogales E. (2001). The binding conformation of Taxol in beta-tubulin: A model based on electron crystallographic density. Proc. Natl. Acad. Sci. USA.

[22] Fu Y., Li S., Zu Y., Yang G., Yang Z., Luo M., Jiang S., Wink M., Efferth T. (2009). Medicinal chemistry of paclitaxel and its analogues. Curr. Med. Chem..

[23] Lowe J., Li H., Downing K.H., Nogales E. (2001). Refined structure of alpha beta-tubulin at 3.5 A resolution. J. Mol. Biol..

[24] (2005). InisghtII Version 2005.

[25] Robinson M.W., McFerran N., Trudgett A., Hoey L., Fairweather I. (2004). A possible model of benzimidazole binding to beta-tubulin disclosed by invoking an inter-domain movement. J. Mol. Graph. Model.

[26] Geney R., Sun L., Pera P., Bernacki R.J., Xia S., Horwitz S.B., Simmerling C.L., Ojima I. (2005). Use of the tubulin bound paclitaxel conformation for structure-based rational drug design. Chem. Biol..

[27] Rodríguez-Menchaca A.A., Navarro-Polanco R.A., Ferrer-Villada T., Rupp J., Sachse F.B., Tristani-Firouzi M., Sánchez-Chapula J.A. (2008). The molecular basis of chloroquine block of the inward rectifier Kir2.1 channel. Proc. Natl. Acad. Sci. USA.

[28] Zhang X., Hu Y., Yuan Z. (2008). Computational analyses of JAK1 kinase domain: subtle changes in the catalytic cleft influence inhibitor specificity. Biochem. Biophys. Res. Commun..

[29] Beccati D. (2009). Investigations of Prebiotics and of Inter- and Intra-Molecular Glycan-Protein Interactions.

[30] Yang Z.W., Yang G., Zu Y.G., Fu Y.J., Zhou L.J. (2009). The conformational analysis and proton transfer of the neuraminidase inhibitors: A theoretical study. Phys. Chem. Chem. Phys..

[31] Yang Z., Nie Y., Yang G., Zu Y., Fu Y., Zhou L. (2010). Synergistic effects in the designs of neuraminidase ligands: Analysis from docking and molecular dynamics studies. J. Theor. Biol..

[32] Wu X.M., Zu Y.G., Yang Z.W., Fu Y.J., Zhou L.J., Yang G. (2009). Temperature-controlled molecular dynamics studies on the folding mechanism of the tubulin active peptides. Acta Phys. Chim. Sin..

[33] Yang G., Wu X.M., Zu Y.G., Yang Z.W., Fu Y.J., Zhou L.J. (2009). Molecular dynamic simulations on the folding and conformational insights of the truncated peptides. J. Theo. Comput. Chem..

[34] Jorgensen W.L., Chandrasekhar J., Madura J.D., Impey R.W., Klein M.L. (1983). Comparison of simple potential functions for simulating liquid water. J. Chem. Phys..

[35] Mitra A., Sept D. (2008). Taxol allosterically alters the dynamics of the tubulin dimer and increases the flexibility of microtubules. Biophys. J..

[36] (2005). Affinity User Guide.

[37] Adelman S.A., Doll J.D. (1976). Generalized Langevin equation approach for atom/solid-surface scattering: General formulation for classical scattering off harmonic solids. J. Chem. Phys..

[38] Mooberry S.L., Weiderhold K.N., Dakshanamurthy S., Hamel E., Banner E.J., Kharlamova A., Hempel J., Gupton J.T., Brown M.L. (2007). Identification and characterization of a new tubulin-binding tetrasubstituted brominated pyrrole. Mol. Pharmacol..

